# Editorial: Information sharing in multiteam systems operating in risky and uncertain environments

**DOI:** 10.3389/fpsyg.2022.1053815

**Published:** 2022-10-07

**Authors:** Sara Waring, Neil Shortland, Sallie J. Weaver

**Affiliations:** ^1^Department of Psychological Sciences, University of Liverpool, Liverpool, United Kingdom; ^2^School of Criminology and Justice Studies, University of Massachusetts Lowell, Lowell, MA, United States; ^3^Healthcare Delivery Research Program, Health Systems and Interventions Research Branch, National Cancer Institute (U.S.), Bethesda, MD, United States

**Keywords:** information sharing, multiteam systems, extreme environments, risk, teams

Multiteam systems (MTSs) are comprised of two or more teams working toward a shared superordinate goal but with unique subgoals at organizational, team, or individual levels (Mathieu et al., 2001). Membership within MTSs is determined by goal and task interdependencies, which can span specialisms, creating a diverse pool of knowledge and resources (Marks et al., 2001, 2005). MTSs can be scaled up or down depending on the task and situation, making them ideal for operating in risky and uncertain environments such as disaster response (Waring et al., 2018), military operations (DeCostanza et al., 2014), and healthcare (Jones et al., 2019). However, research also highlights problems with information sharing within these complex structures, including sharing too little or too much, and using terminology that is difficult to understand across the whole MTS (Mathieu et al., 2001; DeCostanza et al., 2014; Shuffler et al., 2015; Waring et al., 2018). Furthermore, the complexity of the contexts and *ad hoc* nature in which large MTSs often form make it difficult to develop traditional facilitators associated with effective information sharing, such as familiarity (Ren and Argote, 2011), trust (Jarvenpaa and Keating, 2011), and a shared understanding of who knows what (Heavey and Simsek, 2015). Consequently, MTS members often struggle to know what information to share, with whom, and why.

Elsewhere, academic research and public inquiries have highlighted the negative consequences arising from poor information sharing. These include inaccurate and outdated situation awareness, decision errors and delays, cognitive overload, inability to distinguish relevant cues, and hampered team performance (Rencoret et al., 2010; Schraagen et al., 2010; Patrick, 2011; Pollock, 2013; Kerslake, 2018; Waring et al., 2020). However, less focus has been directed toward examining underlying mechanisms that facilitate and hinder information sharing in extreme environments or related intervention approaches. Most MTS research uses experimental methods to study small MTSs with narrow goals and specializations responding to short tasks (Cobb, 1999; Marks et al., 2005; Davison et al., 2012; Bienefeld and Grote, 2014; Firth et al., 2015). In contrast, MTSs operating in extreme environments tend to be larger, more complex and varied in shape and size (Luciano et al., 2015). MTSs in extreme environments must also operate faster and over a longer period of time (Waring et al., 2018, 2019). This has led to questions being raised regarding the extent to which findings from traditional MTS research apply to large MTSs operating in extremis (Shuffler et al., 2015; Waring, 2019).

Accordingly, the goal of this Research Topic is to present research that bridges the methodological gap between the laboratory and the real world. Paying greater attention to examining MTSs operating in a range of risky and uncertain contexts provides opportunities to understand what works in practice to facilitate information sharing processes, along with how they emerge and change over time (Luciano et al., 2015; Shuffler and Carter, 2018; Waring, 2019). The five articles presented within this Research Topic focus on MTSs operating in a diverse range of domains, including public health emergency response (Black et al.), military handling of time sensitive targets (Hærem et al.), long duration space exploration (Verhoeven et al.), offender management (Waring et al.), and a comparison across failed MTS responses to 40 events spanning a range of extreme environments (Campbell et al.). Whilst all five studies draw on subject matter experts rather than naïve participants, various methods are used, reflecting differences in theory development and prior research focus across domains.

Within this Research Topic, qualitative approaches are used in several papers to better understand information sharing practices in domains that have received little prior focus within the MTS literature, allowing cross-validation of theory. This includes Black et al., who examine what non-technical skills are important for improving information sharing and coordination in public health outbreaks. Findings highlight the dynamic nature of public health response and fluid formal and informal MTS memberships, requiring continual investment in relationship building to facilitate information sharing and coordination. In contrast, Verhoeven et al. explore how NASA spaceflight teams work together in high-risk contexts. Findings highlight additional information sharing and coordination challenges as missions become longer and more uncertain, requiring increased investment in pre-flight activities such as cross-functional training to facilitate shared knowledge of roles, responsibilities, and information requirements. Waring et al. examine the causes of information sharing difficulties in offender management and the impact of a new initiative that allows probation services direct access to police systems to view information relevant to their role. Findings highlight that whilst providing direct access can improve the relevance and timeliness of information, this does not address trust issues causes by limited knowledge of the legalities of sharing sensitive information.

In contrast, experimental methods are used to test theoretical assumptions regarding information sharing practices in contexts receiving greater previous focus. Hærem et al. use time-sensitive military simulations to examine the impact of communication setting on development of situation awareness. Findings highlight that ability to share and integrate information to project future status of a situation decreases when co-located teams become distributed. Finally, whilst previous MTS research often takes a static snapshot of end performance in individual domains, Campbell et al. conduct a temporal historiometric analysis of cases across emergency response, commercial transportation, military, and business contexts to determine patterns of within- and between- team behaviors of failing MTSs. Findings highlight a tendency to over engage in within-team information sharing, alignment, and acting behaviors to the detriment of between-team, monitoring, or recalibrating behaviors.

Overall, this body of research contributes to developing knowledge regarding what mechanisms facilitate and hinder information sharing within complex MTSs operating in extremis. It is a testament to the dedication of the researchers continuing to produce studies within this domain during a pandemic that prevented face-to-face research and challenged the ability of community partners to prioritize research partnerships. As we move away from public health restrictions, further focus on methods for observing MTSs within extreme environments will be beneficial for developing temporal theories of inter-team information sharing and improving practice.

## Author contributions

SW wrote the article. NS and SJW edited the article and provided feedback on content. All authors contributed to the article and approved the submitted version.

## Conflict of interest

The authors declare that the research was conducted in the absence of any commercial or financial relationships that could be construed as a potential conflict of interest.

## Publisher's note

All claims expressed in this article are solely those of the authors and do not necessarily represent those of their affiliated organizations, or those of the publisher, the editors and the reviewers. Any product that may be evaluated in this article, or claim that may be made by its manufacturer, is not guaranteed or endorsed by the publisher.
